# Randomized Clinical Trial on Efficacy of Empagliflozin Versus Sitagliptin, In Addition to Metformin in Type 2 Diabetic Patients

**DOI:** 10.7759/cureus.31699

**Published:** 2022-11-20

**Authors:** Muaz Mubashir, Mazhar Ahmed, Hassan Atique, Ahmed Wassan, Mehdi Naqvi, Muneeb Ullah

**Affiliations:** 1 Internal Medicine, Federal Government Polyclinic Hospital Islamabad, Islamabad, PAK; 2 Internal Medicine/Gastroenterology, Federal Government Polyclinic Hospital Islamabad, Islamabad, PAK; 3 General Surgery, Maroof International Hospital, Islamabad, PAK

**Keywords:** randomized trial, glycated hemoglobin (hba1c), sitagliptin, metformin therapy, empagliflozin, diabetes mellitus type 2

## Abstract

Introduction

Diabetes mellitus is a syndrome affecting more than 28.7 million people worldwide and its prevalence in Pakistan is reported to be about 11%. Management includes lifestyle changes and varied therapeutic regimens. Metformin (MET) alone and in combinations is considered as an important agent for glycemic control. Our study is based on MET combination therapy with empagliflozin versus sitagliptin in order to achieve glycemic control.

Methods

This randomized clinical trial was conducted in the Department of Medicine and Allied of Federal Government Polyclinic Hospital, Islamabad, from January 2022 till June 2022. The ethical approval letter numbered FGPC. 1-1/2022/Ethical Committee was taken before the commencement of the trial. The patients were divided into group A and group B. All patients were given MET 1000mg twice a day. Group A patients were additionally given sitagliptin 50mg twice daily whereas Group B patients were additionally given empagliflozin 10mg once daily. Glycemic control was documented with HbA1c at the start of treatment and after three months of treatment in both groups. A proforma was used to collect data. Analysis of the data was performed using the Statistical Package for the Social Sciences version 17 (SPSS Inc., Chicago, USA).

Results

A total of 126 patients were included in the study with a mean age of 53.53 ± 6.49. 81.7% were males while 18.3% were females. The mean reduction in HbA1c from baseline in group A was -0.81 ± 0.19% and in group B was -1.13 ± 0.24% with statistically significant p-value (p-value = 0.000).

Conclusion

Empagliflozin in combination with metformin is more efficacious in maintaining glycemic control as compared to sitagliptin in combination with metformin.

## Introduction

Diabetes mellitus (DM) has been a rising public health problem with increasing prevalence and many complications [1,2]. Sedentary lifestyle and obesity have led to increase in the number of diabetics worldwide which is expected to double in the next 25 years [3]. Its prevalence in Pakistan is reported to be about 11% and is estimated to reach 15% of the total population by 2030 [4,5]. Management of DM includes lifestyle and dietary changes alongside medications. The majority of type 2 DM (T2DM) patients do not achieve ideal diabetic control with metformin (MET) alone and need additional medications [6]. In individuals with hemoglobin A1c (HbA1c) of 7.5% to 9%, guidelines recommend starting dual treatment with MET and one additional medication. Dual treatment is also considered in cases where initial HbA1c is less than 7.5% but has not improved in three months of treatment. Research trials for the best second-line agent to use with MET are still in process with no consensus as yet [7,8]. Sodium-glucose cotransporter-2 (SGLT2) inhibitors represent a newer class of anti-diabetic medicines that cause a reduction in renal tubular glucose reabsorption, producing a reduction in blood glucose without stimulating insulin release [9,10]. This results in the lowering of postprandial and fasting glucose levels, as well as lowering body weight through secondary caloric loss by excreting glucose in urine. Empagliflozin is SGLT2 inhibitor that is available in the oral form [11]. Empagliflozin is shown to be effective as a monotherapy as well as in combination with additional oral anti-diabetic medications in various randomized controlled studies [12-14]. Sitagliptin is a competitive inhibitor of the enzyme dipeptidyl peptidase 4 (DPP-4). It degrades gastrointestinal hormone (GIP) and glucagon-like peptide 1 (GLP-1), released in response to meal thus stimulating insulin release while suppressing glucagon release from alpha cells of the pancreas by inhibiting the breakdown of the GIP and GLP-1. SGLT-2 inhibitors are considered to reduce cardiovascular disease-related morbidity and mortality in diabetic patients. Empagliflozin in particular was statistically superior to sitagliptin in terms of lowering the risk of cardiovascular-related death [15].

## Materials and methods

This randomized clinical trial was conducted in the Department of Medicine and Allied of Federal Government Polyclinic Hospital, Islamabad, from January 2022 till June 2022. The ethical approval letter numbered FGPC. 1-1/2022/Ethical Committee was taken before the commencement of the trial from the Federal Government Polyclinic Hospital. A simple random sampling technique was used and a total of 126 patients were enrolled after obtaining informed consent. Patients above the age of 18 years diagnosed with T2DM and taking metformin for more than three months were included in the study. Patients with a history of chronic liver or renal disease, chronic diarrhea, pregnancy, lactation, drug abuse, poor compliance to medications, incomplete data and those who lost for follow-up were excluded from the study. The patients were divided into two groups using the lottery method, group A and group B, each containing 63 patients. All patients were given MET 1000mg twice a day. Group A patients were additionally given sitagliptin 50mg twice daily whereas Group B patients were additionally given empagliflozin 10mg once daily. Glycemic control was documented with HbA1c at the start of treatment and after three months of treatment in both groups. A proforma was used to collect data including age, gender, group (group A or group B), duration of T2DM, HbA1c and weight at presentation and after three months. Patient confidentiality was maintained. Analysis of the data was performed using the Statistical Package for the Social Sciences version 17 (SPSS Inc., Chicago, USA). The frequency and percentage for qualitative variables included efficacy and gender. Standard deviation and mean were calculated for the age of the patients. Stratification was used to affect modifiers such as age and gender. Post-stratification chi-square test was used. Its value of less than 0.05 with a 95% confidence interval was considered statistically significant.

## Results

A total of 126 patients were included in the study, 81.7% were males while 18.3% were females. Details of gender distribution and the mean age in years of patients in group A and group B are shown in Table 1.

**Table 1:** Demographics parameters of the study

Particulars		Group A (Sitagliptin + Metformin)	Group B (Empagliflozin + Metformin)
Gender	Total		
Male	103	60	43
	81.7%	95.2%	68.3%
Female	23	3	20
	18.3%	4.8%	31.7%
Age in years	Mean ± standard deviation	51.83 ± 6.30	55.24 ± 6.27

The mean duration of T2DM in group A was 4.35 ± 1.44 years and in group B was 4.33 ± 1.32 years with a p-value of 0.949. The mean value of HbA1c and weight at presentation and after three months in respective groups are shown in Table 2.

**Table 2:** Mean HbA1c and weight in the groups kg: kilogram HbA1c: Hemoglobin A1c

Variables	Groups	Mean	SD
Weight at presentation (kg)	Group A	67.41	6.79
	Group B	71.71	6.79
Weight after Three Months (kg)	Group A	64.11	6.82
	Group B	64.98	8.82
HbA1c at presentation (%)	Group A	8.87	0.40
	Group B	8.65	0.41
HbA1c after Three Months (%)	Group A	8.05	0.45
	Group B	7.52	0.47

The mean reduction in HbA1c from baseline in group A was -0.81 ± 0.19% and in group B was -1.13 ± 0.24% with a statistically significant p-value (p-value = 0.000). Additionally, mean reduction in weight between the groups was also significant with group B having more impact (p-value = 0.000) as shown in Table 3.

**Table 3:** Reduction in weight and HbA1c between groups

Variables	Groups	Mean	p-value
Weight change from presentation (kg)	Group A	-3.30	0.000
	Group B	-6.73	
HbA1c change from presentation (%)	Group A	-0.81	0.000
	Group B	-1.13	

## Discussion

Male predominance was seen in our study with male to female ratio of 4.47 comparable to other studies within Pakistan [16,17]. However, there are studies that report female predominance [18,19]. Mean age in our study ranged approximately between 51 to 56 years that correlates with national and international data [20,21]. The mean duration of T2DM in both groups was at least four years. Patients often remain in denial at initial diagnosis and often try home treatments, homeopathic and hakeemic medications before they finally present to a medical facility. Additionally, symptoms are ignored at the initial stage and finally when it becomes bothersome enough, patients visit their respective specialists. The mean value of HbA1c in our study ranged from 8.6 to 8.9 at presentation comparable to international data [22-24]. The treatment was effective in both groups but group B showed more reduction in mean HbA1c value and mean weight as compared to group A. This means that using empagliflozin in combination with MET has better efficacy as compared to sitagliptin in combination with MET. The efficacy of empagliflozin and MET combination in reducing HbA1c levels, lowering body weight and arterial blood pressure without causing hypoglycemia has already been documented in different studies [25,26]. Additionally, it is reported that as an initial therapy especially in coronary vascular disease patients, it shows better symptomatic improvement is seen [27,28]. This has led to an increased usage of this combination as an initial therapy. Moreover, now researches and trials on single-pill combination are in process to enhance the convenience and compliance of patients [25,28]. Trials have demonstrated that combining empagliflozin with MET improves long-term HbA1c levels when compared to MET alone [29].

Our study has multiple limitations since it is a single-centered study with a short duration. The sample size is also small compared to the type of study. Mean weight is not a good comparative parameter and body mass index should have been more appropriate outcome measure. The medication brands, dosage and compliance should have also been standardized. It is nevertheless a pilot study and further research can be performed based on this to reach more conclusive outcomes and recommendations in our region for initial T2DM medication regimes. The cross-relation of groups with co-morbid conditions like hypertension, ischemic heart disease, kidney disease and liver disease might have added additional insight to the study.

## Conclusions

Empagliflozin in combination with metformin is more efficacious in maintaining glycemic control as compared to sitagliptin in combination with metformin. Additionally, this combination is safe in coronary vascular disease patients and aids in weight reduction.
